# Application of classical and novel integrated machine learning models to predict sediment discharge during free-flow flushing

**DOI:** 10.1038/s41598-022-23781-x

**Published:** 2022-11-12

**Authors:** Fahime Javadi, Kourosh Qaderi, Mohammad Mehdi Ahmadi, Majid Rahimpour, Mohamad Reza Madadi, Amin Mahdavi-Meymand

**Affiliations:** 1 Department of Water Engineering, Shahid Bahonar University of Kerman, Kerman, Iran; 2 Department of Water Engineering, University of Jiroft, Jiroft, Iran; 3 Institute of Hydro-Engineering, Polish Academy of Sciences, Warsaw, Poland

**Keywords:** Hydrology, Engineering, Mathematics and computing

## Abstract

In this study, the capabilities of classical and novel integrated machine learning models were investigated to predict sediment discharge (Q_s_) in free-flow flushing. Developed models include Multivariate Linear Regression (MLR), Artificial Neural Network (ANN), Adaptive Neuro-Fuzzy Inference System (ANFIS), Group Method of Data Handling (GMDH), and four hybrid forms of GMDH and Support Vector Regression (SVR) in combination with Henry Gas Solubility Optimization (HGSO) and Equilibrium Optimizer (EO) algorithms. The number of 160 datasets that were applied to assess these models was extracted from the Janssen (PhD’s Thesis, Engineering, Civil and Environmental Engineering. University of California, 1999) experimental study. Input parameters to predict Q_s_ included the water level in the reservoir (h_w_), bed level in the flushing channel (h_b_), outflow (Q_out_), inflow (Q_in_), and elapsed time of flushing (T). The performance of all models was evaluated by four statistical indices of root mean square error (RMSE), mean absolute error (MAE), correlation coefficient (R^2^), and Mean absolute relative error (MARE). Evaluation of results demonstrated that the HGSO and EO algorithms could enhance the accuracy of the GMDH model (up to 26% and 22% in terms of RMSE), respectively. According to statistical criteria, the SVR-EO and SVR-HGSO provided the highest accuracy in both training (R^2^ = 0.98) and validation phases (R^2^ = 0.96). Moreover, among the developed models, the GMDH-HGSO algorithm provided excellent fitness to the observed data (R^2^ = 0.96, RMSE = 22.37, MAE = 15.65, and MARE = 0.26). The results indicated the high efficiency of the HGSO and EO algorithms in improving the accuracy of the GMDH and SVR models. However, among the developed models, the GMDH-HGSO is the most accurate model and is recommended for sediment transport modelling.

## Introduction

Dams are large hydraulic structures used for several purposes, such as water supply for drinking and agriculture, industrial usage, flood control, navigation and hydropower generation. These structures alter the sediment balance of natural rivers. A dam reservoir significantly reduces the flow velocity, acts as a sediment trap, and causes deposition of a portion of the incoming sediment load. While fine sediments are transported into the reservoir, the coarse materials are deposited immediately at the head of the reservoir to form the delta deposits^1^.

Sediment deposition reduces and eventually eliminates the reservoir storage volume. Loss of reservoir storage volume causes the reduction of reservoir function and the dam’s practical lifetime, resulting in economic losses. According to the literature, annually, 0.5–1% of the global water storage volume is lost by sediment deposition. In addition, the losing annual global storage volume is higher than the volume of newly constructed reservoirs^2^. The construction of new dams is complicated due to the environmental restrictions, construction and design costs, and the unique and scarce appropriate locations^3^. Management of existing reservoirs to control the progressive deposited sediments and long-term use of reservoirs is necessary, which needs the knowledge of sedimentation processes in reservoirs and dredging methods^4^.

Measures for reducing the long-term deposited sediments in the reservoirs can be divided into three general categories: reducing the sediment inflow from the upstream, diverting the sediments route to minimize deposition, and removing the accumulated sediment in reservoirs. Lowering the sediment inflow from the upstream is divided into two main categories: 1: reducing the sediment particles (soil and channel erosion control at the source) and 2: trapping the sediment particles upstream of the reservoirs. Sediment route diverting is a set of techniques to bypass the sediments around the reservoirs or pass through tunnels or canals. Deposited sediments can be removed by flushing and mechanical removal methods. The sediment management strategies depend on many factors, including the climate, the reservoir operation, the water supply needs, and the catchment and reservoir characteristics. Therefore, a sustainable sediment management strategy for a unique reservoir does not exist. Among different management strategies for controlling reservoirs' sedimentation, flushing is an efficient hydraulic technique for storage capacity restoration and conservation. In the reservoirs, hydraulic flushing is classified into pressure flushing and drawdown flushing (free-flow sediment flushing)^3^.

Machine learning models are flexible and non-parametric algorithms that can make a connection between the inputs and outputs variables without a deep knowledge of the physical behaviour of the system. Successful performances of machine learning models have attracted the attention of many researchers. For instance, Emamgholizadeh et al. reported the superiority of Artificial Neural Networks (ANN) to Adaptive Neuro-Fuzzy Inference System (ANFIS) in predicting the geometry of flushing half-cone^5^. Li et al. reported the suitable performance of the Back Propagation training Artificial Neural Network (BP-ANN) to analyze the relationship between the sediment flushing efficiency of the Three Gorges Reservoir (TGR)^6^. Cao et al. compared the results of a novel SVR- Henry Gas Solubility Optimization (SVR-HGSO) model with the SVR, SVR-FA, SVR- Particle Swarm Optimization (SVR-PSO), SVR- Ant Lion Optimizer (SVR-ALO), SVR-Dragonfly Algorithm (SVR-DA) and SVR- Salp Swarm Algorithm (SVR-SSA) algorithms. They found that the SVR-HGSO algorithm outperformed other algorithms^7^. Qaderi et al. used hybrid models based on the Group Method of Data Handling (GMDH) to predict bedform dimensions of alluvial channels. They concluded that hybrid models perform better than ordinary GMDH and empirical equations^8^. EL Bilali et al. evaluated the performance of the ANN and a modified universal soil loss equation coupled with multiple linear regression (MUSLE-MLR) to predict yearly sedimentation in the reservoir. They found that the performance of the ANN model is higher than other methods in predicting of yearly sedimentation in the reservoir^9^. Qaderi et al. compared the performance GMDH based models to predict daily dew point and reported that GMDH-HS and GMDH-SCE models produced better results than other developed models in predicting the dew point temperature^10^. Qaderi et al. used support vector machine (SVM), ANFIS, ANN, gene-expression programming (GEP), and integrated GMDH with harmony search (HS), and shuffled complex evolution (SCE) algorithms to predict bridge pier scour depth. The results indicated the superiority of ANFIS to other developed models^11^. Zeynoddin et al. checked out generalized linear stochastic model (GLSM) result with ANFIS, ANN, GEP, and SVM tuned by the firefly algorithm (SVM-FA) to forecast the weekly and monthly lake water levels. The results revealed that the GLSM model has higher accuracy than other models^12^. EL Bilali et al. compared the performance of SVR, K-nearest neighbour (K-NN), random forest (RF), and ANN models in groundwater level prediction. They proved that the ANN is the most accurate non-linear model^13^. Sayari et al. evaluated the ability of ANFIS, GMDH, multi-layer perceptron neural network (MLPNN), support vector regression (SVR), multivariate linear regression (MLR), and integrate these models with the firefly algorithm (FA) to predict infiltrated water volume in furrow irrigation. The results demonstrated the superiority of the MLPNN-FA and SVR-FA models^14^. Roy et al. developed Equilibrium Optimizer-based Extreme Learning Machine (EO-ELM) for rainfall-runoff modelling. The EO-ELM model was compared with a Deep Neural Network (DNN) with ELM, Kernel ELM (KELM), particle swarm optimization-based ELM (PSO-ELM), SVR, ANN, and Gradient Boosting Machine (GBM). The Results demonstrated that the EO-ELM was the most accurate model^15^. Mahdavi-Meymand et al. applied different kinds of GMDH that integrated with PSO and HGSO algorithms to simulate the maximum hydro-suction dredging depth. The results demonstrated that the GMDH-HGSO algorithm provides an excellent fit to the observed data^16^. Ezzaouini et al. used RF, adaptive boosting (AdaBoost), SVR, K-NN, and ANN models to predict the suspended sediment load. Results showed that all models have good accuracy in predicting the daily suspended sediment load^17^.

Many researchers have studied the reservoir behaviour during sediment flushing which include field and experimental research. According to the authors’ knowledge, machine learning models have not been used to model free-flow flushing channel formation, which highlights this study. In this study, the capability of machine learning models (MLR, MLPNN, GMDH, ANFIS, GMDH-HGSO, GMDH-EO, SVR-HGSO, and SVR-EO) is compared in the prediction of free-flow flushing channel formation. Another highlight of this study is the integration of two new meta-heuristic algorithms (EO and HGSO) with SVR and GMDH. These novel models are analyzed exclusively in this study.

## Methodology

### Data collection

In this study, in total, 160 datasets were used to investigate and compare the ability of classical and novel integrated machine learning models in predicting Q_s_ during free-flow flushing. The datasets were extracted from the Janssen^18^ experimental study report. Janssen^18^ investigated the effect of reservoir drawdown on flushing channel formation. Experiments were performed in a concrete flume 50 m in length, 2.4 m wide and 1.5 m high. Sediments used in these experiments were non-cohesive sediment with mean grain size (D_50_) and sediment saturated density of 1.25 mm and 1270 kg/m^3^, respectively. Deposits were set 10 cm above the valve gate threshold and were paved with side slopes of 1%. Water levels in the reservoir, bed levels in the flushing channel, width of the flushing channel, outflow, and sediment discharge were measured over time to investigate the variations of flushing channel characteristics along the reservoir.

The water level in the reservoir (h_w_), bed level in the flushing channel (h_b_), outflow (Q_out_), inflow (Q_in_), and elapsed time of flushing (T) are considered as input parameters to predict sediment discharge (Q_s_). 112 datasets (70%) were used for training the developed models, 24 datasets (15%) were considered as validation data to prevent overtraining, and 24 datasets (15%) were used to evaluate the accuracy of the implemented models. A summary of the statistical criteria of utilized datasets is illustrated in Table 1.

**Table1:** Statistical characteristics of data.

Description	Notation	Parameters	Minimum	Maximum	Mean	Standard deviation
Water levels (m)	h_w_	X_1_	0.026	0.087	0.052	0.0167
Bed levels (m)	h_b_	X_2_	0.091	0.135	0.110	0.0105
Outflow (m^3^/s)	Q_out_	X_3_	0.0011	0.0114	0.004	0.0021
Inflow(m^3^/s)	Q_in_	X_4_	0.0007	0.0082	0.0033	0.0025
Time (min)	T	X_5_	5	50	22.28	10.84
Sediment discharge (g/s)	Q_s_	Y	1.7	956	109.00	133.43

Moreover, all the variables (inputs and output) before the training process were normalized, as follows:

1 $${X}_{i}^{^{\prime}}=\frac{{X}_{i}-{X}_{min}}{{X}_{max}-{X}_{min}},$$

where the maximum and minimum values of variables are denoted by ${X}_{min}$ and ${X}_{max}$, respectively.

### Conventional machine learning models

#### Group method of data handling (GMDH)

The GMDH neural network is a basic technique of self-organizing data mining that was introduced by Ivakhnenko^19^ as a rival to the stochastic approximation method^20^. Proposed algorithm is based on a multi-layer structure that provides a structure for simulating and modelling complex phenomena, image processing, and data mining. A complicated discrete function called Ivakheneco polynomial is used to connect inputs and output variables in the GMDH.

In the current study, a polynomial function was used as a transfer function in the neurons of the middle and output layers as follows:

2 $$Y={W}_{0}+{W}_{1}{X}_{1}+{W}_{2}{X}_{2}+{W}_{3}{X}_{1}^{2}+{W}_{4}{X}_{2}^{2}+{W}_{5}{X}_{1}{X}_{2},$$

where *W* is the coefficients’ vector (network weights), *X* is the input vector, and *Y* is the output. In the conventional GMDH, the coefficients are determined by the least square estimation (LSE) model^21^.

#### Support vector regression (SVR)

SVR generally is an extended version of support vector machines (SVM). SVR was developed by Vapnik^22^ to solve the regression problems by the SVM model. The SVR model uses the structural risk minimization technique to find the best regression hyperplane, which is defined by the following equation:

3 $$Y=\vartheta \varphi \left(z\right)+c.$$

In this equation, *Y* denotes the non-linear regression function to predict the target vector,$\vartheta$ is the weight vector, and *φ(z)* is an irregular higher dimension mapping input data. The coefficients *c* and $\vartheta$ are estimated as follows:

4 $$Minimize :{R}_{reg}=\left[\frac{1}{2}{\Vert \vartheta \Vert }^{2}+P\sum_{i=1}^{n}{(\xi }_{i}+{\xi }_{i}^{*})\right],$$

5 $$Subject\, to: \left\{\begin{array}{c}{Y}_{i}-\left(\vartheta \varphi \left({z}_{i}\right)+{c}_{i}\right)\le \varepsilon +{\xi }_{i}\\ \left(\vartheta \varphi \left({z}_{i}\right)+{c}_{i}\right)-{Y}_{i}\le \varepsilon +{\xi }_{i}^{*}\\ {\xi }_{i},{\xi }_{i}^{*}\ge 0\end{array}\right.,$$

where *P* factor is the regularization cost parameter, $\varepsilon$ is the acceptable error margin, and ${\xi }_{i},{\xi }_{i}^{*}$ are positive constants called slack variables^23^.

#### Adaptive neuro-fuzzy inference system (ANFIS)

ANFIS is a hybrid model developed based on the Takagi–Sugeno (TS) fuzzy inference system^24^. ANFIS has both neural network (e.g., ability to train machines, the ability of parallel processing, and connectionist structures) and fuzzy logic (e.g., simplicity and flexibility, high speed of training, and can be combined easily) advantages in a single framework. This structure makes the ANFIS a robust model for formulating non-linear problems and forecasting time series phenomena. ANFIS uses a fuzzy inference system (FIS) to calibrate a membership structure or parameters with a combination of methods like the least-squares-type method and the back-propagation algorithm. ANFIS structure comprises five layers and each layer includes different nodes. These layers include the fuzzy layer, production layer, normalization layer, de-fuzzy layer, and total output layer. The first layer consists of several membership functions that convert input variables to fuzzy inputs. The second layer is formed when fuzzy rules are determined for the nods. In the third layer, the effectiveness of the second layer outputs is normalized by rules. The fourth layer receives as input the normalized values from the third layer and performs the defuzzification process. The fifth layer (the last layer), utilizes the defuzzification values returned by the fourth layer to produce the system output as a numerical variable^25^.

#### Artificial neural networks (ANNs)

The ANNs are one of computational intelligence systems composed of many units or nodes. These nodes are called neurons. Neurons are connected by weights and are aggregated into separated layers. The multi-layer perceptron neural network (MLPNN) is a class of feed-forward ANNs that consist of three layers: the input layer (introduction of data to the network), the multiple hidden layers (data processing), and the output layer (results from data processing). Except for input neurons, each neuron uses a non-linear activation function of the weighted summation of the inputs of the previous layer. The structure of this model is highly influenced by the problem variables, and the interconnection strategy is used in the training step^14^.

#### Multiple linear regression (MLR)

Multiple linear regression is the development of simple linear regression for cases with more than one variable. Basic equation for this model is:

6 $$Y={\beta }_{0}+{\beta }_{1}{X}_{1}+{\beta }_{2}{X}_{2}\dots {\beta }_{k}{X}_{k}+\varepsilon ,$$

where *Y* is the output, *X* is the input vector, *β* represents the regression coefficient vector, and *ε* is the standard estimation error^26^.

### Meta-heuristic algorithms

#### Henry gas solubility optimization (HGSO)

HGSO is a new meta-heuristic optimization method imitating Henry’s law that was presented by Hashim et al.^27^. According to the Henry’s law, the amount of dissolved gas in a liquid is proportional to its partial pressure above the liquid. This law was formulated by William Henry as the following equation:

7 $${C}_{g}={H}_{g}\times {P}_{g},$$

where, *C*_*g*_ is the solubility of the gas, *P*_*g*_ denotes the gas partial pressure, and *H*_*g*_ is Henry’s constant. Henry’s law constants depended on the system temperature.

The HGSO algorithm consists of the following steps: initialization process (the positions of the population are initialized), Clustering (to divide the population into equal clusters), evaluation (the clusters are evaluated to determine the gas that reaches the highest equilibrium), updating Henry’s coefficient and solubility of gas, updating the positions and escaping from the local optimum (selecting and re-initializing a number of worst agents). A more detailed presentation of the HGSO algorithm can be found in Hashim et al.^27^.

#### Equilibrium optimizer (EO)

EO is a new swarm-based meta-heuristic algorithm proposed by Faramarzi et al.^28^. EO is inspired by the control volume mass balance models. Search agents in EO are particles (solution). Concentration is considered as the particle’s position. Positions of search agents are randomly updated based on the best current solutions (equilibrium candidates) until the equilibrium state (optimal result) is achieved. EO, like other stochastic optimization algorithms, considers an initial population to start the optimization process. Positions of the population are randomly initialized in the space search. At the beginning of the optimization process, the particles are classified to specify the equilibrium candidates. Equilibrium candidates are evaluated to provide a search pattern for the particles. These candidates are five particles that are nominated as the equilibrium candidates. Four equilibrium candidates have the best fitness in all populations, and another is chosen based on the average fitness. The four best particles improve the exploration, while the average one helps to increase the exploitation abilities of the algorithm. The populations can be determined based on the type of problems^28^.

### Integrated machine learning models

**Figure 1:**
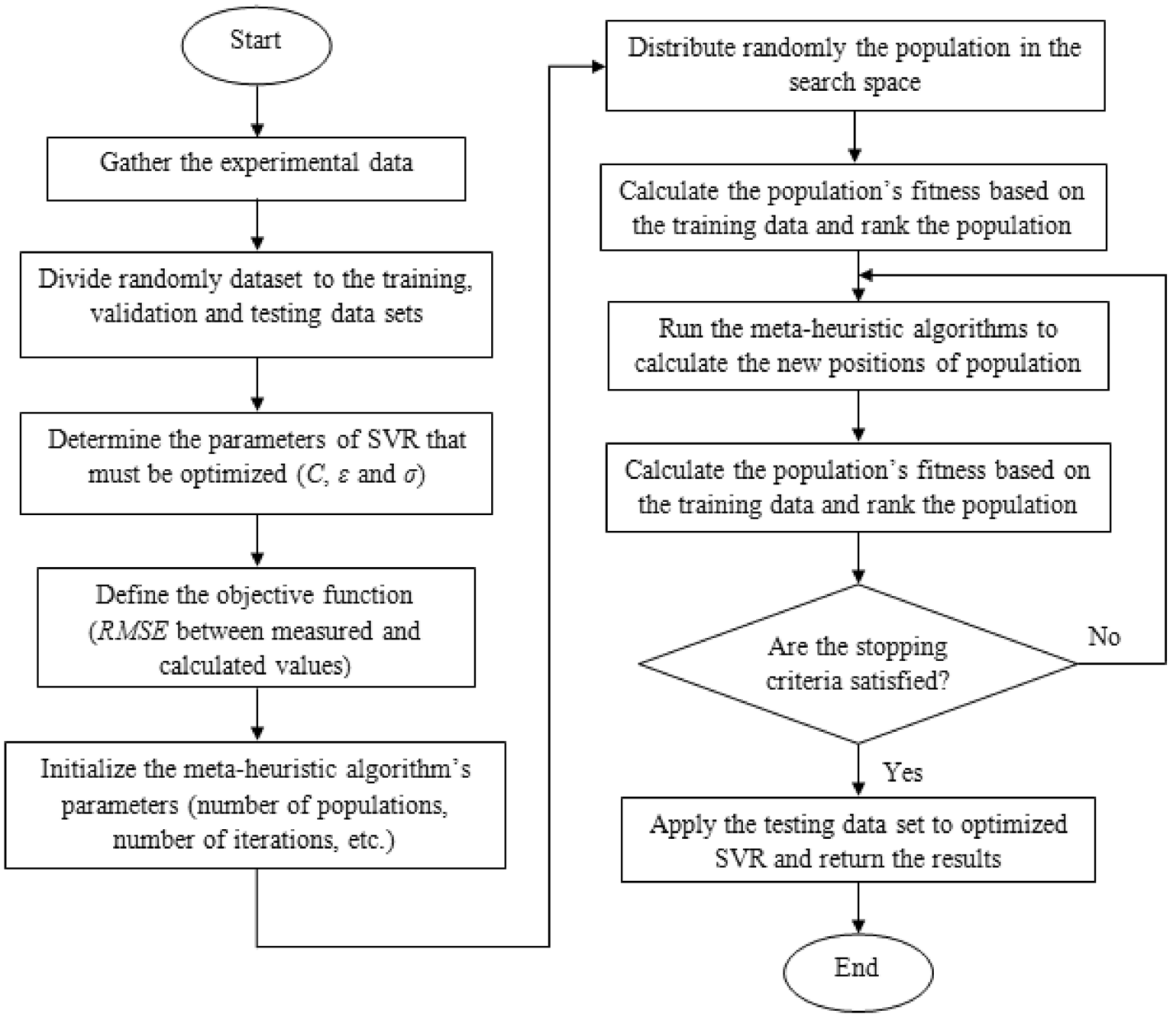
SVR- HGSO and SVR- EO flowchart.

**Figure 2:**
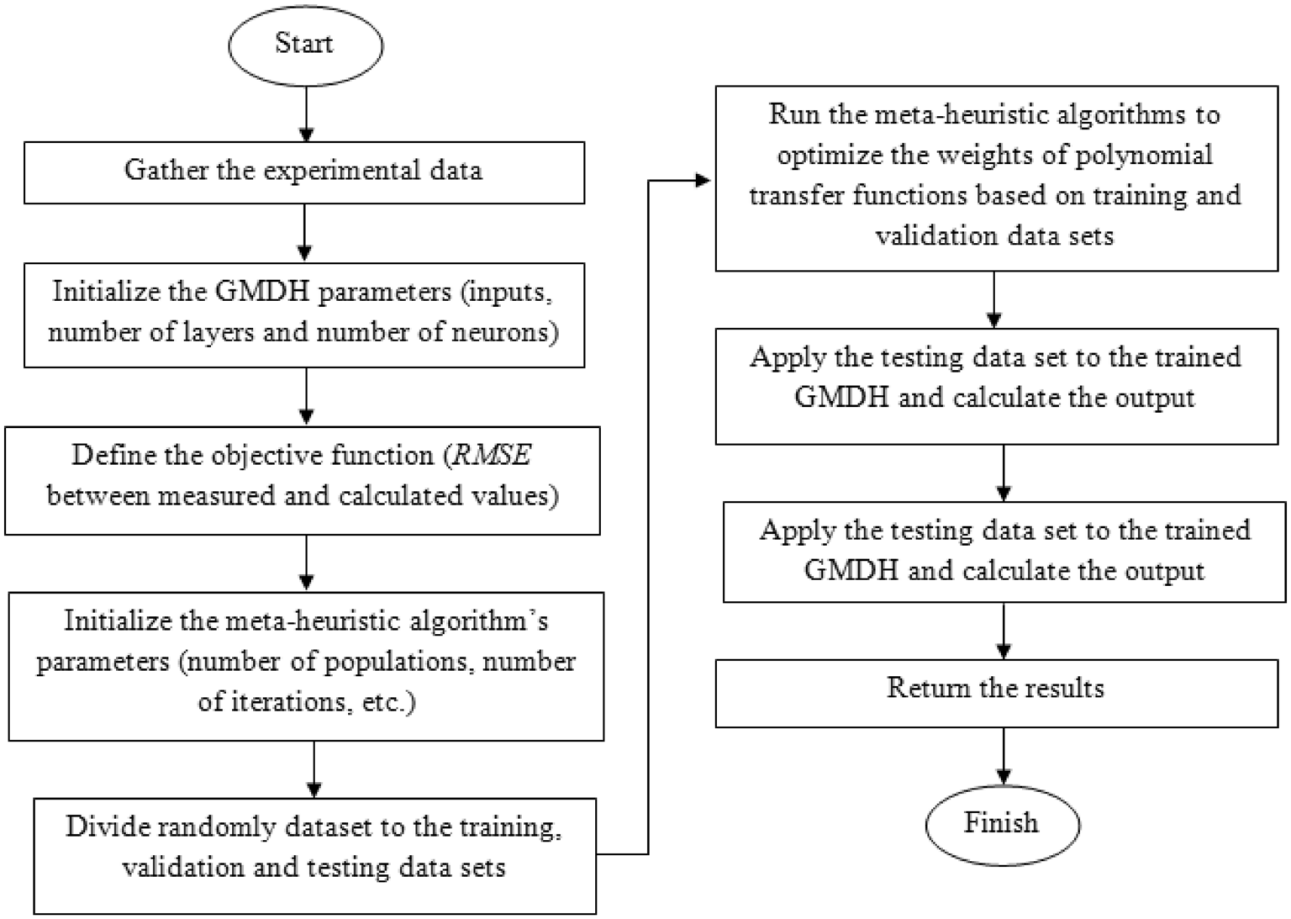
GMDH- HGSO and GMDH- EO flowchart.

Meta-heuristic algorithms are robust optimization strategies for low and high dimension complex problems. Meta-heuristic algorithms in machine learning models optimize the structure, and calibrate unknown weights coefficients of machine learning models or both structure and weights. In this study, HGSO and EO were applied to optimize the weights of GMDH and unknown coefficients of SVR. A GMDH with two middle layers and maximum of ten neurons for each layer was considered. Weights of quadratic polynomial transfer functions of neurons were obtained by EO and HGSO. Regularization parameter (*C*), the insensitive loss coefficient (*ε*), and the kernel constant (*σ*) are three parameters of SVR that affect the output results. HGSO and EO were used to obtain the optimal values of these parameters. Figures 1 and 2 illustrate the flowcharts of integrated GMDH and SVR models, respectively. HGSO and EO, like most other meta-heuristic algorithms, have some parameters that must be initialized before the optimization process. In this study, the HGSO and EO parameters values were initialized based on the main source study^27,28^. The values of the initial parameters of meta-heuristic algorithms are shown in Table 2.

**Table 2:** Considered initial values of developed EO and HGSO algorithms for this study.

Algorithm	Parameter	Value
EO	SVR population	50
	GMDH population	100
	Iterations	100
	a_1_ (is a constant in EO equations)	2
	a_2_ (is a constant in EO equations)	1
	Generation Probability (GP)	0.5
HGSO	SVR population	50
	GMDH population	100
	Number of clusters	2
	Iterations	100
	l_1_ (is a constant in HGSO equations)	0.005
	l_2_ (is a constant in HGSO equations)	100
	L_3_ (is a constant in HGSO equations)	0.01
	a and β	1

### Evaluation criteria

The results of developed models are evaluated by four standard statistical criteria. These statistical indices are included the root mean square error (RMSE), mean absolute error (MAE), correlation coefficient (R^2^), and mean absolute relative error (MARE). RMSE is used to indicate the difference between predicted and observed values. MAE is the average of absolute differences between predicted and observed values and is indifferent to the direction of errors. The model will have the best prediction if the values of RMSE and MAE are close to zero^29^. The correlation coefficient measures the degree of similarity between predicted and measured data. R^2^ = 1 corresponds to a perfect model match to the observed data. Predicted and actual values are similar if the MARE value is close to 0^30^. These indexes were calculated as follows:

8 $$MARE=\frac{1}{n}\sum_{i=1}^{n}\left(\frac{\left|{X}_{Oi}-{X}_{Pi}\right|}{{X}_{Oi}}\right),$$

9 $$MAE=\frac{1}{n}\sum_{i=1}^{n}\left|{X}_{Pi}-{X}_{Oi}\right|,$$

10 $$RMSE=\sqrt{\frac{1}{n}\sum_{i=1}^{n}{({X}_{Pi}-{X}_{Oi})}^{2}},$$

11 $${R}^{2}=\frac{\sum_{i=1}^{n}({X}_{Pi}-{\overline{X} }_{P}){(X}_{Oi}-{\overline{X} }_{O})}{\sqrt{\sum_{i=1}^{n}{({X}_{Pi}-{\overline{X} }_{P})}^{2}{{(X}_{Oi}-{\overline{X} }_{O})}^{2}}},$$

where the observed and predicted values are denoted by ${X}_{Oi}$ and ${X}_{Pi}$, respectively. The ${\overline{X} }_{O}$ and ${\overline{X} }_{P}$ represent the mean of the observed and predicted values, and n is the number of observations.

## Results

In this paper, the capability of several new-developed machine learning models was investigated in free-flow flushing sediment discharge modelling. The performance of each model was evaluated by standard statistical criteria of MARE, RMSE, MAE, and R^2^ for both the training and testing phases. Simulation results of the training, validation, and testing datasets are presented in Table 3.

**Table 3:** Statistical indices of the proposed model in the training, validation and testing datasets.

Model	Training dataset				Validation dataset				Testing dataset			
	RMSE (g/s)	R^2^	MAE (g/s)	MARE	RMSE (g/s)	R^2^	MAE (g/s)	MARE	RMSE (g/s)	R^2^	MAE (g/s)	MARE
MLR	55.11	0.86	39.74	1.78					53.10	0.77	40.09	0.75
MLPNN	16.66	0.99	12.92	0.31	40.41	0.82	26.85	0.33	36.59	0.87	26.13	0.51
ANFIS	26.35	0.97	16.77	0.57	34.76	0.91	25.58	0.3	31.35	0.89	24.49	0.49
GMDH	33.09	0.95	25.00	0.64	30.82	0.90	21.13	0.25	30.35	0.92	21.02	0.38
GMDH-HGSO	42.99	0.92	29.72	0.41	38.51	0.88	22.44	0.2	22.37	0.96	15.65	0.26
GMDH-EO	37.10	0.94	26.24	0.42	36.98	0.90	24.27	0.24	23.71	0.95	18.35	0.26
SVR-HGSO	21.27	0.98	12.28	0.43	18.54	0.96	14.58	0.17	26.65	0.93	16.48	0.21
SVR-EO	20.35	0.98	11.84	0.31	18.78	0.96	14.61	0.17	26.21	0.93	16.34	0.21

**Figure 3:**
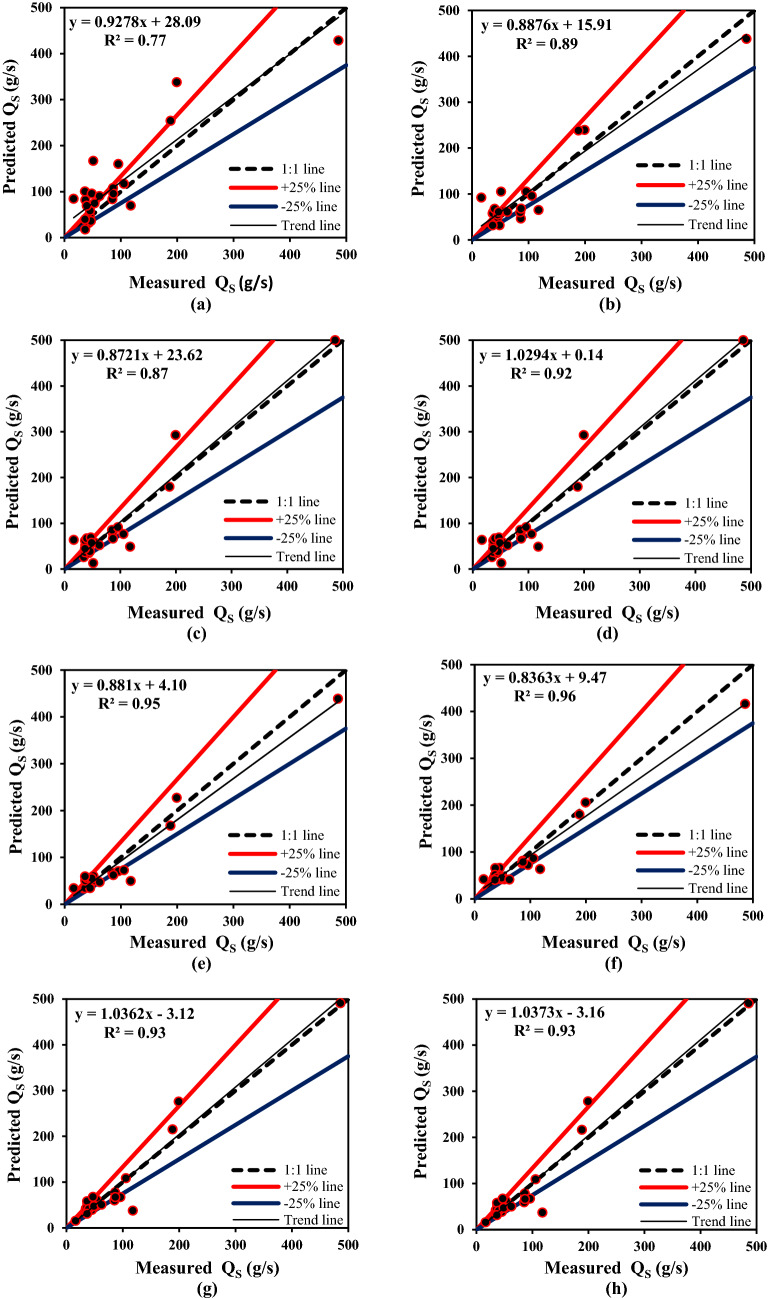
Scatter plots of the measured and predicted Q_s_ in the testing phase. (**a**) MLR; (**b**) ANFIS; (**c**) MLPNN; (**d**) GMDH; (**e**) GMDH-EO; (**f**) GMDH-HGSO; (**g**) SVR-EO; (**h**) SVR-HGSO.

It can be observed from Table 3 that the R^2^ values of all models are higher than 0.8, which means all applied models have good performance during both the training and validation phases. In the training phase, the MLPNN, SVR-EO, and SVR-HGSO models produced the highest R^2^, which are 0.99, 0.98, and 0.98, respectively. But in the validation set, two models, SVR-EO and SVR-HGSO, have the highest R^2^ (0.96, 0.96, respectively). This shows the suitable accuracy of SVR-EO and SVR-HGSO models in predicting of sediment discharge in training and validation sets. In hybrid GMDH-based models, it is observed that the GMDH had better performance compared to the integrated versions of GMDH (GMDH-HGSO and GMDH-EO) during both the training and validation phases. RMSE values of GMDH-HGSO and GMDH-EO are 42.99 g/s and 37.10 g/s in the training dataset and 38.51 g/s and 36.98 g/s in the validation dataset, while in GMDH, it is 33.09 g/s and 30.82 g/s, respectively. However, in the testing phase, GMDH-HGSO and GMDH-EO models are better than GMDH. It can be observed that in the training, validation, and testing datasets, the MLR model obtained very high values of RMSE and low R^2^ values among all the applied models. In other words, there is no linear relation between the variables of the free-flow flushing phenomena. Comparison between GMDH and ANFIS indicates that their obtained results are very similar in the testing phase. However, in the training phase, ANFIS is slightly more accurate than GMDH in terms of R^2^, RMSE, MAE, and MARE. On the other hand, for predicting Q_s_ in the testing phase, GMDH-HGSO obtained the best R^2^, RMSE, and MAE statistics of 0.96, 22.37, and 15.65, respectively. GMDH-EO is in the second rank. Values of R^2^, RMSE, and MAE of GMDH-EO in the testing phase are 0.95, 23.71, and 18.35, respectively. SVR-EO and SVR-HGSO are slightly better than GMDH-HGSO and GMDH-EO in terms of MARE. The performance of the GMDH-EO model was slightly better than SVR-EO and SVR-HGSO. Generally, the performances of GMDH-HGSO, GMDH-EO, SVR-EO, and SVR-HGSO are better than other models in the testing phase. This indicates the ability of HGSO and EO algorithms in the optimal determination of the GMDH coefficients and SVR parameters. Figure 3 presents the scatter plots of predicted values of Q_s_ using the developed models in the testing phase. The lines − 25% and + 25% represent two dispersion bounds, while the 1:1 line denotes the line of agreement.

**Figure 4:**
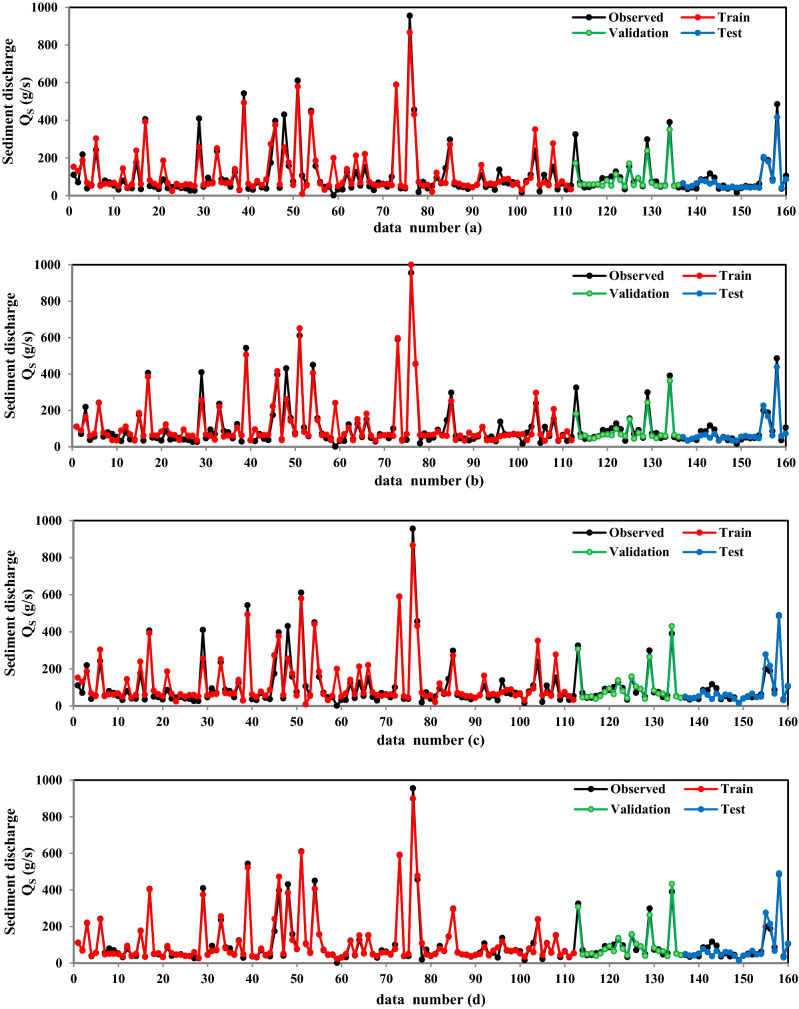
Performance of the best models in sediment discharge prediction in the training, validation, and testing phases (**a**) GMDH-HGSO; (**b**) GMDH-EO; (**c**) SVR-HGSO; (**d**) SVR-EO.

The data points located under the 1:1 line reveal the under-prediction of applied models. Moreover, the over-predicted data are situated on top of the 1:1 line. As it can be seen from Fig. 3, except for the MLR model, the most scatter points of the applied models are between the − 25% and + 25 lines. According to Fig. 3, about 75% of the scatter points of GMDH-HGSO and 71% of GMDH-EO, SVR-EO and SVR-HGSO are located within a ± 25% error from the 1:1 line (correct agreement line). Moreover, 58% of the predicted values of the GMDH model are located in this area. It can be concluded that using the HGSO and EO algorithms improves prediction accuracy. It can be concluded from Fig. 3 and Table 3, among new-developed machine learning models, GMDH-HGSO, GMDH-EO, SVR-EO, and SVR-HGSO models demonstrated acceptable performance. GMDH-HGSO is the best model for predicting sediment discharge in the free-flow flushing channel. The performance of the best models in predicting sediment discharge in the training, validation, and testing phases are illustrated in Fig. 4.

**Figure 5:**
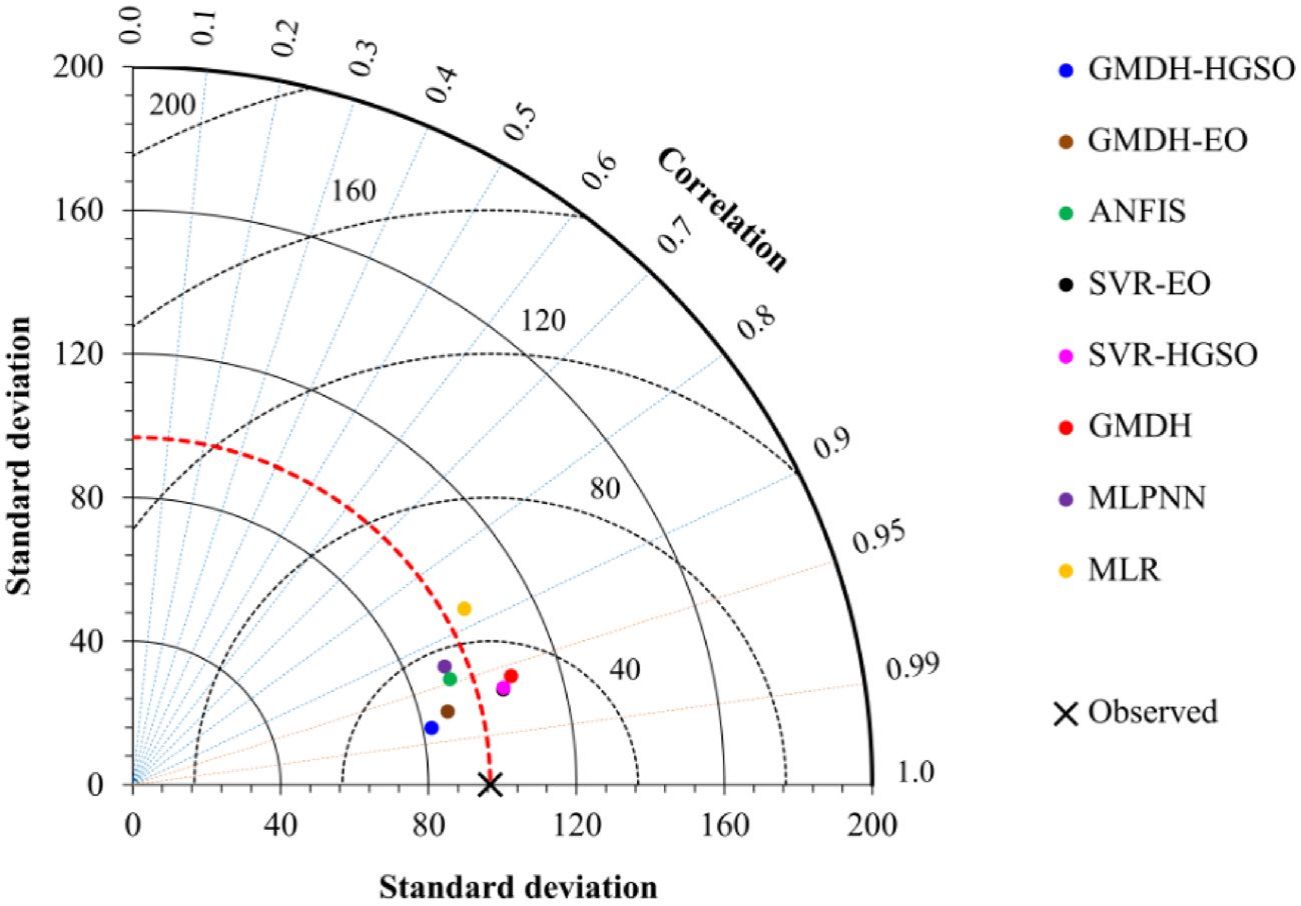
Taylor diagram of the applied models (testing data).

Taylor’s diagram is a mathematical diagram to evaluate and compare the performances of different models using the Pearson correlation coefficient, the central root-mean-square error (CRMSE), and the standard deviation. The distance between each model and the observed point is a measure to evaluate the models’ performance. If the model point and observed point are similar, the Pearson correlation coefficient is close to 1, the CRMSE is close to 0, and the calculated standard deviation for both of them is the same^31^. Figure 5 illustrates the Taylor diagram of developed models. According to the results presented in Fig. 5, all machine learning models have relatively high correlation and low CRMSE which indicates the excellent performance of the applied models. The MLR has a low pattern correlation. The GMDH and the hybrid forms of GMDH and SVR have relatively small differences with observed values. However, The GMDH-HGSO model with relatively high correlation and low CRMSE is the most accurate method.

## Discussion

Reservoir sedimentation represents a serious threat to water management, flood control, and energy production that should be investigated. Classical and novel integrated models can be valuable tools for sediment management. In this study, several models, including MLR, ANN, ANFIS, GMDH and four hybrid forms of GMDH and SVR in combination with HGSO and EO algorithms were used to predict the sediment discharge (Q_s_) in free-flow flushing. The results revealed that these models have the acceptable performance to predict the sediment discharge in the free-flow flushing. The findings of this study confirm that the non-linear models’ accuracy is higher than linear methods.

The models in this study were trained based on the experimental data of Janssen^18^. Whether the models were trained based on data with the sufficient domain or not is a challenging question. In an actual project where the maximum water level during free flushing is around 10 m the scale based on the Janssen^18^ experiments and Froude number would calculate 115. With this scale, the maximum discharges in the prototype would calculate 1617 m^3^/s which is realistic. However, training machine learning methods with more data and broader ranges of parameters would develop more advanced models.

Meta-heuristic algorithms are robust tools for optimizing complicated problems. Sediment transport is a complex hydraulic phenomenon. The results of this study approved that using novel algorithms such as HGSO and EO increases the accuracy of machine learning performance. The application of new integrative machine learning models is recommended to simulate other hydraulic problems. Also designing new experiments with large scales or field measurements to increase the domain of data is a good topic for the future direction of study.

## Conclusions

Free-flow flushing is an efficient method for reducing deposited sediments in reservoirs. Accurate prediction of free-flow flushing parameters is significantly essential for the economical design of reservoirs and sediment management. In this study, new developed integrated machine learning models were applied to predict the sediment discharge of free-flow flushing (Q_s_). Models used included MLR, ANFIS, MLPNN, GMDH, and the integration of GMDH and SVR with EO and HGSO. Performances of advanced machine learning models are close to each other. However, the GMDH-HGSO is the most accurate model. Results revealed that the HGSO and EO algorithms are robust optimization tools for training machine learning models. The HGSO and EO improved the GMDH performance by about 5% and 3%, respectively. The application of the GMDH-HGSO model in reservoir management for predicting the sediment output is recommended. However, increasing the domain of parameters and analyzing the models’ performance with new data set is a good topic for the future direction of study.

## Data Availability

The utilized data are available in Janssen (1999), and by the Corresponding author per request.
